# Intravenous immunoglobulin treatment improves multiple neuropsychiatric outcomes in patients with pediatric acute-onset neuropsychiatric syndrome

**DOI:** 10.3389/fped.2023.1229150

**Published:** 2023-10-16

**Authors:** Jelena Eremija, Sanjay Patel, Sydney Rice, Michael Daines

**Affiliations:** ^1^ Division of Allergy, Immunology and Rheumatology, Department of Pediatrics, University of Arizona, Tucson, AZ, United States; ^2^ Department of Internal Medicine, Arizona College of Osteopathic Medicine, Midwestern University, Glendale, AZ, United States; ^3^ Division of Developmental and Behavioral Pediatrics, Department of Pediatrics, University of Arizona, Tucson, AZ, United States

**Keywords:** intravenous immunoglobulins (IVIG), pediatric acute-onset neuropsychiatric syndrome (PANS), pediatric autoimmune neuropsychiatric disorders associated with streptococcal infections (PANDAS), neuropsychological testing, immunomodulation, immune deficiency

## Abstract

Pediatric Acute-Onset Neuropsychiatric Syndrome (PANS) is defined by acute onset of diverse neuropsychiatric manifestations, presumably in the setting of underlying immune dysfunction**.** We used standardized neuropsychological testing to assess how intravenous immunoglobulins (IVIG) impact neurological and cognitive functions in PANS patients by comparing pretreatment with post-treatment scores. A 5-year retrospective study was undertaken in Children's Postinfectious Autoimmune Encephalopathy Center at University of Arizona. We identified 12 children diagnosed with PANS and treated with immunomodulatory IVIG doses, who also completed neuropsychological testing before and after treatment. We tracked multiple patient characteristics, type/timeline of testing, and number of IVIG courses. Score change of 1 standard deviation in any tested domain/subdomain was considered improvement. We further reviewed records for laboratory signs of triggering infection and immune dysfunction. Improvement occurred in 11/12 patients, in one or multiple domains/subdomains, independently of time between disease onset and IVIG initiation (0–7 years). Participants received 1–7 IVIG courses. Improvement was primarily seen in memory (58%), sensory-motor (37%) and visual-motor integration (30%). In 5/12 patients we detected hypogammaglobulinemia requiring ongoing IVIG replacement, one patient had isolated low IgA. Only one patient had to discontinue IVIG therapy due to severe adverse effects. Standardized neuropsychological testing represents an important tool to objectively measure improvement in PANS patients. IVIG was tolerated well and showed efficacy in the vast majority of participants, independently from timelapse since disease onset, emphasizing impact of immunomodulation in PANS. Significant presence of baseline hypogammaglobulinemia in children with PANS emphasizes the presumed role of immune dysfunction in disease pathogenesis.

## 1. Introduction

Pediatric autoimmune neuropsychiatric disorder associated with streptococcal infections (PANDAS) was initially identified in 1990s (1) but a new and broadened definition was later established in an attempt to capture wider spectrum of neuropsychiatric disorders: “pediatric acute-onset neuropsychiatric syndrome” or PANS (2). The PANS diagnostic criteria included (1) acute onset of obsessive-compulsive disorder (OCD) or severely restricted eating, (2) concomitant manifestation of additional neuropsychiatric symptoms, with similarly dramatic onset (i.e., anxiety, depression, aggressive/oppositional behaviors, developmental regression, worsening school performance, sensory or motor dysfunction, sleep, or continence disorders) in (3) absence of other medical and neurological conditions (2). These varied and expanded features of PANS and PANDAS have been confirmed by other groups over time (3–5). Etiology of this multifaceted disorder is still not fully elucidated, but there is a growing body of data, especially in the past decade, suggesting that abnormal immune system function might play a significant role in this illness (6–10). Diagnosis of PANS remains challenging due to the complex presentation, relapsing-remitting course, and unclear etiology of the disorder. In 2015, guidelines for assessment were published (4). Given the multitude of symptoms and still uncertain etiology, treatment of PANS remains challenging. Guidelines were published in 2017 including behavioral (11), immunomodulatory (12) and infection prevention (13) to help navigate therapeutic endeavors.

Evaluation of immunomodulatory interventions in patients with PANS continues to be of great importance, given that underlying immune dysfunction is thought to have a significant impact on PANS development. Intravenous immunoglobulins (IVIG) have been investigated in randomized controlled trials (14, 15) which overall favored IVIG compared to placebo. More recently, an open label study (6) showed unequivocal benefit of immunomodulatory IVIG therapy in children with PANS, with statistically significant reduction of symptoms, including OCD, overall clinical severity and parental concerns. Additionally, some studies have shown increased patient/parent satisfaction following IVIG therapy indirectly suggesting improved patient outcomes (16).

Given limited, yet reassuring data regarding efficacy of IVIG immunomodulation in PANS patients, we aimed to expand existing knowledge on this topic by providing objective quantifiable assessment of outcomes following IVIG treatment by comparing pre- and post-treatment neuropsychological scores.

## 2. Methods

A retrospective record review was undertaken in Children's Postinfectious Autoimmune Encephalopathy (CPAE) Center of Excellence at The University of Arizona, following Institutional Review Board (IRB) approval. We evaluated patients with a PANS diagnosis who were assessed in CPAE Center from 2017 to 2022. Besides diagnosis of PANS, further inclusion criteria consisted of (1) treatment with immunomodulatory IVIG doses and (2) completion of neuropsychological testing before and after treatment. We excluded patients who did not require IVIG therapy or did not complete both pre- and post-treatment neuropsychological evaluation.

Patients’ characteristics such as age, gender, age at PANS onset, state of residence and applied therapeutic interventions were noted through record review. We additionally investigated records of children included in the study for any laboratory determinants of immunodeficiency or other immune dysfunction, as well as reported structural or inflammatory changes on brain magnetic resonance imaging (MRI) and cerebrospinal fluid (CSF) analyses.

All patients met criteria for diagnosis of PANS corresponding to PANS Consensus criteria (4). The decision to start patients on IVIG therapy was made based on ongoing or worsening symptoms, in spite of treatment with other therapeutic anti-infectious and/or anti-inflammatory modalities: non-steroidal anti-inflammatory drugs (NSAIDs), antibiotics (therapeutic or immunomodulatory/prophylactic) and systemic steroids.

In order to better evaluate risks/tolerability of IVIG immunomodulation, we tracked dosing and number of IVIG courses, as well as any reported adverse events.

### 2.1. Neuropsychological assessments

Testing of patients was completed by licensed clinical psychologists (located in the area of patient's residence), who were not blinded to patient's treatment course. A variety of standardized testing batteries appropriate for neuropsychological testing in children and adolescents were used. This included Kaufman Brief Intelligence Test, 2nd edition (KBIT-2) as a stand-alone test early in the development of the clinic, as well as other more comprehensive assessments. Comprehensive intellectual and cognitive tests included The Wechsler Abbreviated Scale of Intelligence, 2nd edition (WASI-2) and Wechsler Intelligence Scale for Children, 4th or 5th edition (WISC-4 or 5). Memory was tested via Wide Range Assessment of Memory and Learning, 2nd edition (WRAML-2) and California Verbal Learning Test Children's (CVLT-C). The Wide Range Achievement Test 4th or 5th edition (WRAT-4 or 5), and Wechsler Individual Achievement Test 3rd edition (WIAT-3) were used as a tool for assessing overall learning achievements. Visual-motor integration was evaluated via Beery-Buktenica Developmental Test of Visual-Motor Integration (Beery VMI). Additionally, visual motor, visual spatial and fine motor skills were assessed by Wide Range Assessment of Visual Motor Abilities (WRAVMA) Pegboard.

Type of neuropsychological testing was noted as well as delay between the 1st IVIG course to pre-and post-test, and scores were compared for each patient prior and after treatment, if the same testing tool was used in both assessments. For the purpose of this retrospective study, authors chose to define improvement as positive change of 1 standard deviation in any tested area or a sub-section of the test.

### 2.2. Analyses

Descriptive statistical methods were used to calculate, analyze, and present data.

## 3. Results

Through retrospective record review, we identified 12 consecutive patients, all of which qualified for diagnosis of relapsing-remitting PANS and who were treated with immunomodulatory doses of intravenous immunoglobulins (Table 1). Half of the patients were Arizona residents. There was a slight female predominance of 58% in our cohort. Laboratory signs of active streptococcal infection (throat swab during the suspected inciting illness) or prior exposure [elevated Antistreptolysin-O (ASO) titers] were noted in 7 patients, of which 2 had other potential triggers/exposures in the similar time frame: one with influenza infection detected in multiple family members and one with pertussis in grandmother. The remaining 5 patients had symptoms of infectious illnesses without a recognized microbial agent (not all were tested for streptococcal infections when symptomatic). The average age for PANS diagnosis was 8.5 years with mean delay from diagnosis to IVIG treatment being 3 years, although almost half (5/12) of the patients received IVIG within the 2 years of diagnosis. The maximum delay between PANS diagnosis and IVIG therapy was 7 years in 1 patient. These delays were consequences of different reasons, some of which included late diagnosis, worsening in severity/control of symptoms on other treatments, and occasionally happened prior to evaluation in our CPAE Center at The University of Arizona. The majority of the patients were treated with 3 consecutive monthly courses of IVIG at 2 g/kg each. The number of treatments varied between 1 and 7 courses which depended on experienced adverse events-leading to decreased number of courses, or, in case of continued improvement in patients with severe symptoms-additional courses.

**Table 1:** Patient characteristics.

Patient	Gender	Age Dg/Tx	Streptococcus^a^	Pre-test delay^b^	Post-test delay^b^
Patient 1	F	9/16	No	28	69
Patient 2	M	13/14	Yes	133	91
Patient 3	F	7/9	No	102	218
Patient 4	M	2/8	No	144	52
Patient 5	M	12/14	Yes	−6	210
Patient 6	F	2/7	Yes	29	41
Patient 7	M	5/9	Yes	13	80
Patient 8	F	11/12	Yes	238	80
Patient 9	M	16/16	No	42	45
Patient 10	F	4/10	Yes	16	47
Patient 11	F	12/13	Yes	54	55
Patient 12	F	9/10	No	76	204

Age Dg/Tx: age (years) at the time of diagnosis and initiation of intravenous immunoglobulin treatment.

^a^ Based on positive throat swab and/or Antistreptolysin-O titers.

^b^ Time frame between pre-treatment or post-treatment neuropsychological testing (days) from the start of the 1st IVIG course.

Only one patient had to discontinue treatment due to severe adverse effects (AEs), as he developed symptoms suggestive of aseptic meningitis with the first dose of IVIG. An additional 3 patients reported milder AEs: 2 patients had headaches, and one nausea, none of which were serious enough to require discontinuation of treatment.

Patients qualified for IVIG treatment based on failure to improve on one or multiple other treatment modalities, such as anti-infectious and/or anti-inflammatory medications: NSAIDs, antibiotics (therapeutic or immunomodulatory/prophylactic) and systemic steroids. Eleven patients (92%) were unsuccessfully managed with all three types of treatment (antibiotic, NSAID and prednisone) prior to starting IVIG courses, whereas the remaining 1 child received antibiotic and steroid. These treatments were not continued during IVIG therapy. Patients were also treated with a variety of psychiatric and other medications, as well as non-pharmacological interventions prior to (and continued in parallel) with IVIG, as depicted in Table 2. We are aware that these interventions are very important for many aspects of disease control. However, we believe that IVIG had the largest impact on the improvements in areas of neuropsychological functioning that were focus of this study given that the patients still suffered from notable PANS signs and symptoms in spite of all other used treatments, prior to receiving IVIG. Authors are unaware of any relevant external factors that would have had notable influence on reported recovery.

**Table 2:** Therapeutic interventions prior to initiation of IVIG.

	Pre-IVIG PANS treatments	Additional pharmacologic interventions	Non-pharmacologic interventions
Patient 1	Abx, NSAIDs, Steroids	None	None
Patient 2	Abx, NSAIDs, Steroids	Guanfacine, SSRI	CBT, IEP
Patient 3	Abx, NSAIDs, Steroids	SSRI	Counseling
Patient 4	Abx, NSAIDs, Steroids	Lisdexamfetamine, SSRI	BT, 504 Plan, OT, ST
Patient 5	Abx, NSAIDs, Steroids	SSRIs, Atomoxetine, Amphetamine/dextroamphetamine	IEP, Counseling
Patient 6	Abx, NSAIDs, Steroids	Methylphenidate	504 Plan
Patient 7	Abx, NSAIDs, Steroids	None	None
Patient 8	Abx, NSAIDs, Steroids	SSRIs, Hydroxyzine, Aripiprazole, Trazodone	IEP, Counseling
Patient 9	Abx, NSAIDs, Steroids	SSRIs	BT, Counseling
Patient 10	Abx, Steroids	None	IEP, ABA, ST, OT
Patient 11	Abx, NSAIDs, Steroids	SSRI, Aripiprazole	CBT, Counseling
Patient 12	Abx, Nsaids, Steroids	Clonidine, Lisdexamfetamine	None

Abx, antibiotics; NSAIDs, non-steroidal anti-inflammatory drugs; steroids, prednisone 1 mg/kg for 5 days used in majority of patients; SSRI(s), selective serotonin reuptake inhibitor(s). In case of our patients this included: fluoxetine, fluvoxamine, sertraline, citalopram, escitalopram. IEP, individualized educational plan; ST, speech therapy; OT, occupational therapy; CBT, cognitive-behavioral therapy; BT, behavioral therapy; ABA, applied behavioral analysis.

Following IVIG treatment, the vast majority of the patients experienced some beneficial effects (11/12). Please refer to Table 3 for detailed, individual breakdown of testing that was completed and improvements that were noted. Pretreatment testing was done with a wide range of intervals, averaging 72 days (see Table 1). Post-treatment testing was done on average 99 days after the 1st IVIG course (SD was 69 days) and maximum delay was 218 days in a patient who received 4 IVIG treatments (see Table 1). Two patients were tested with KBIT-2 and both had relevant (+1SD) improvement on post-treatment assessment. The remaining 10 patients were tested with varying combinations of neuropsychological tests to evaluate cognition, memory, intellectual, integrative, and other areas of functioning. Unfortunately, the same combination of assessments was not done in all patients, and in some children different tests were used to assess the same area of functioning before and after treatment. 50% of patients tested with multi-component tests experienced improvement in 2 or 3 of tested areas and another 40% achieved improvement in one of the evaluated domains. Positive effects of IVIG primarily manifested in memory increase (58% or 5/9 tested patients), followed by sensory-motor and visual-motor integration (significant changes noted in 37% and 30% of tested patients, respectively).

**Table 3:** Individual patient results.

	Dg-IVIG delay (years)	Number of IVIG courses	KBIT-2	WASI-2	WISC-4/5	WRAML-2 CVLT-C	WRAT-4/5 WIAT-3	Beery VMI	WRAVMA Pegboard
Patient 1	7	3	n/a	↔	↔	↔	↔	↑	↑
Patient 2	1	3	n/a	↔	n/a	↔	↔	↔	↔
Patient 3	2	4	n/a	↔	↔	↑	↔	↔	↔
Patient 4	6	1	n/a	↔	↔	↑	↔	↔	↔
Patient 5	2	7	n/a	n/a	↔	↑	n/a	↔	n/a
Patient 6	5	2	n/a	n/a	↑	n/a	n/a	↔	n/a
Patient 7	4	3	n/a	↔	↔	↔	↑	↔	↑
Patient 8	1	3	n/a	n/a	↑	↑	↔	↑	↔
Patient 9	0	3	n/a	↑	↔	↑	↔	↔	↔
Patient 10	6	2	n/a	↔	↔	↔	↔	↑	↑
Patient 11	1	3	↑	n/a	n/a	n/a	n/a	n/a	n/a
Patient 12	1	5	↑	n/a	n/a	n/a	n/a	n/a	n/a
Improved	n/a	n/a	2/2	1/7	2/9	5/9	1/8	3/10	3/8

n/a: testing not done, or not comparable, ↑: significantly improved. ↔: no change. Dg-IVIG delay, delay from diagnosis to treatment; KBIT-2, Kaufman brief intelligence test, 2nd edition; WASI-2, Wechsler abbreviated scale of intelligence, 2nd edition; WISC-4/5, wechsler intelligence scale for children, 4th or 5th Edition; WRAML-2, wide range assessment of memory and learning, 2nd edition; CVLT-C, California Verbal Learning Test Children's; WRAT-4/5, Wide Range Achievement Test 4th or 5th edition; WIAT-3, wechsler individual achievement test 3rd edition; Beery VMI, Beery-Buktenica developmental test of visual-motor integration.

Our data did not demonstrate that delay from PANS diagnosis to IVIG treatment negatively impacted the desired effects of immunomodulation, as both patients that received IVIG within 2 years from diagnosis and the ones with longer delay experienced comparable benefits. Interestingly, the only patient who did not experience significant response to IVIG and the two who benefited the most (by showing improvement in 3 areas) all received the treatment within 1 year following the diagnosis of PANS.

We searched the records for any laboratory signs of abnormal immune system function, with remarkable findings of hypogammaglobulinemia in half of the patients: 1 had isolated low IgA and 5 had low IgG requiring continued IVIG replacement.

Brain MRI was completed in five patients prior to initiation of IVIG immunomodulatory treatment and prior to establishing PANS diagnosis, within initial assessment of these patients in order to exclude other causes of disease symptoms. Imaging is not part of PANS evaluation but is commonly done during initial work-up in, although it is not a necessary step in PANS diagnosis. Varied MRI modalities were used, but there were no signs of inflammatory changes in any of the patients who underwent imaging. Five patients underwent lumbar puncture and CSF analysis (prior to being evaluated in our center), all of which showed normal routine cytology and biochemistry. Additionally, two had negative oligoclonal band testing, two had negative encephalitis panels and one had normal levels of CSF immunoglobulins.

## 4. Discussion

Pediatric Acute-Onset Neuropsychiatric Syndrome is a complex set of neuropsychiatric manifestations whose etiology has not yet been fully elucidated. PANS continues to be challenging to diagnose and difficult to treat. Given variable presentation and relapsing-remitting course in some patients (1, 4, 8, 15, 17), objective confirmation and quantification of improvement represent the most important tasks for multidisciplinary teams caring for PANS patients. Furthermore, limited data from scarce randomized controlled trials on IVIG efficacy (14, 15) drive the effort to find ways to objectively evaluate outcomes in order to confidently recommend this therapeutic intervention. Having a tool for precise and unbiased effect measurement would allow for assessments and comparisons of different treatments, thus aiding in data-driven decision making and therapeutic guidelines for this complex disorder.

Recent trial by Melamed et al. (6). successfully demonstrated measurable improvement in 21 patients with PANS who were treated with 6 courses of 1 g/kg/dose IVIG every 3 weeks. In comparison, most patients in our retrospective study received the same cumulative dose of intravenous immunoglobulins, but over 3 courses (though patients received 1–7 courses), also with notable benefits. These two sets of data, in addition to other prior publications that favored IVIG treatment over placebo and were (at least partially) blinded and randomized (14, 15) prove that IVIG is an important and effective therapeutic option for patients with PANS diagnosis. Although a varied number of immunoglobulin courses and doses was used in all of these studies, IVIG still showed efficacy. Furthermore, one could argue that trials by Williams and Perlmutter (14, 15) would have shown higher impact of IVIG on PANS/PANDAS symptoms if multiple courses were used given that cumulative dose in both of these trials was 2 g/kg given over 2 days, and was not repeated. This potentially might allow for some flexibility in treatment dosing and duration and lead to a more personalized approach in tailoring each patient's treatment plan. Additionally, using periodical behavioral and neuropsychological testing consistently may allow for objective monitoring and quantification of progress between IVIG courses and guide decisions on next steps based on measurable outcome assessments.

We chose to use the first dose as the time point for calculating interval to post-treatment testing, for several reasons. Primarily, this decision stems from the fact that the original trials of IVIG therapy in PANS/PANDAS used a single immunoglobulin dose and still showed improvement (14, 15), thus suggesting that this impactful treatment can be effective with even a single dose and that this could potentially be the most effective dose. Furthermore, some of the patients in our cohort got a single dose of IVIG and improved, supporting this notion.

OCD is the most prominent, frequently defining feature of PANS and is the one primarily used as a measure of improvement in PANS patients (6, 14, 15). However, PANS presents a broad range of other psychiatric and neuropsychological manifestations that sometimes even overshadow OCD (1, 8, 15, 17). This is the reason our study focused on additional pertinent neuropsychological disorders commonly seen within PANS spectrum and provided a novel expanded insight into beneficial effects of IVIG comparing to other trials. Furthermore, parental, and overall clinical impressions are also commonly used in these trials as a measure of successful treatment but may be insufficiently specific (such as Clinical Global Impression of Severity) and prone to bias in case of parental questionnaires (6, 14). Another rather subjective outcome measure for PANS treatment that has been reported in literature and favored IVIG over other therapeutic approaches, has been patient satisfaction (16). Overall, our study is complementary to other trials and further adds to the previously recognized positive impact that immunomodulation through IVIG infusions can have on a significant proportion of children suffering from PANS.

Hypogammaglobulinemia was detected in half of the study population, underscoring the importance of immune dysregulation in etiology of Pediatric Acute-Onset Neuropsychiatric Syndrome. Although humoral immunodeficiencies (IDs) are the most common type of ID, this overrepresentation of hypogammaglobulinemia in our cohort might come as a manifestation of previously recognized and well-studied connection between immunodeficient conditions and autoimmunity (18–20), although it may also be a reason these patients were more susceptible to infections preceding onset of PANS. It can also be argued that positive clinical responses of PANS patients to immunomodulatory interventions indirectly prove that immune dysfunction is a pertinent foundation of PANS development. Although the specific causes of hypogammaglobulinemia are not focus of this study, there is no suspicion that it developed secondary to other therapeutics used for management of study participants. Even the systemic steroids were used in the form of short bursts, which do not have significant impact on immune globulin production.

Our study does have some limitations. It is conducted in a single center with a relatively small sample size (not surprising as this is a rare disorder) and very heterogenous population, thus risk-stratification and generalizations cannot be reliably made. Lack of control group prevents us from better assessing exact impact (and statistical significance) of IVIG on PANS improvement, compared to naturally relapsing-remitting course of the disease and any effects of other ongoing pharmacological and non-pharmacological interventions that were used in these patients. The immune system assessment that is presented here is very limited, but this was not the primary focus of the study.

The most prominent flaw of our study is lack of consistency in the timeline and type of testing used, primarily between subjects, but unfortunately in some cases, there were variations in testing used in the same patient before and after IVIG treatment. This limited our ability to extract complete data sets on every patient and led to omission of certain outcomes, potentially resulting in underestimation of IVIG impact.

Uniform and standardized implementation of neuropsychological testing, within our CPAE Center, but also nationally, is needed to allow for further data pooling as well as comparison of outcomes amongst patients and different centers especially given the rarity of this illness.

## Data Availability

The original contributions presented in the study are included in the article/Supplementary Material, further inquiries can be directed to the corresponding author.
